# Synthetic populations of South African urban areas

**DOI:** 10.1016/j.dib.2018.05.126

**Published:** 2018-05-26

**Authors:** Johan W. Joubert

**Affiliations:** Centre for Transport Development, Department of Industrial and Systems Engineering, University of Pretoria, 0002 South Africa

## Abstract

This article presents the procedure followed to generate complete synthetic populations from the South African National Census. The populations are accurate at both household and individual level, and were generated for nine major metropolitan and provincial areas. The disaggregate description of the population is useful in a variety of modelling contexts, especially if one wants to observe or study the distributional effects of, for example, policy measures. That is, studies in which equity and equality are of concern. The datasets are publicly available from https://doi.org/10.17632/dh4gcm7ckb.1.

**Specifications Table**

**Table:** 

Subject area	*Population modelling, Bayesian network*
More specific subject area	*Synthetic population*
Type of data	*Compressed Extensible Markup Language (XML) files*
How data was acquired	*Both input data sources, the South African Census data from 2011, and the 10% public use micro-sample, are publically available. The subplace tables were used as control totals to reweigh a pool of synthetic households that were sampled from Bayesian networks, which in turn was estimated from subsets of the 10% public use micro sample.*
Data format	*Compressed Extensible Markup Language (XML) files*
Experimental factors	*Sampling from the estimated Bayesian networks are random. For each study area one hundred populations were generated, each with a different random seed.*
Experimental features	*This paper deals only with the generation of the data sets with no further experiments conducted on the data.*
Data source location	*Not applicable. Data are randomly generated.*
Data accessibility	*The data prepared as part of this article is publicly available. Due to size limitations only the first two populations for each study area is published on Mendeley:*https://doi.org/10.17632/dh4gcm7ckb.1*. The remainder is available on a public Git repository (URL included with the Mendeley data set).*

**Value of the data**

• A synthetic population allows one to understand and study the underlying structure of a population at a very disaggregate level.
• The provided populations are controlled at the household level using (household) income, and at individual levels using gender and population group. The result provides a complete stock of individuals while accounting for detailed demographic, socioeconomic information, and household structure.
• Synthetic populations for nine major urban areas in South Africa are provided, each accounting for the demographic and socioeconomic diversity of the specific area. This is useful in studying inequality and diversity in multiple contexts.

## 1 Data

**Fig. 1:**
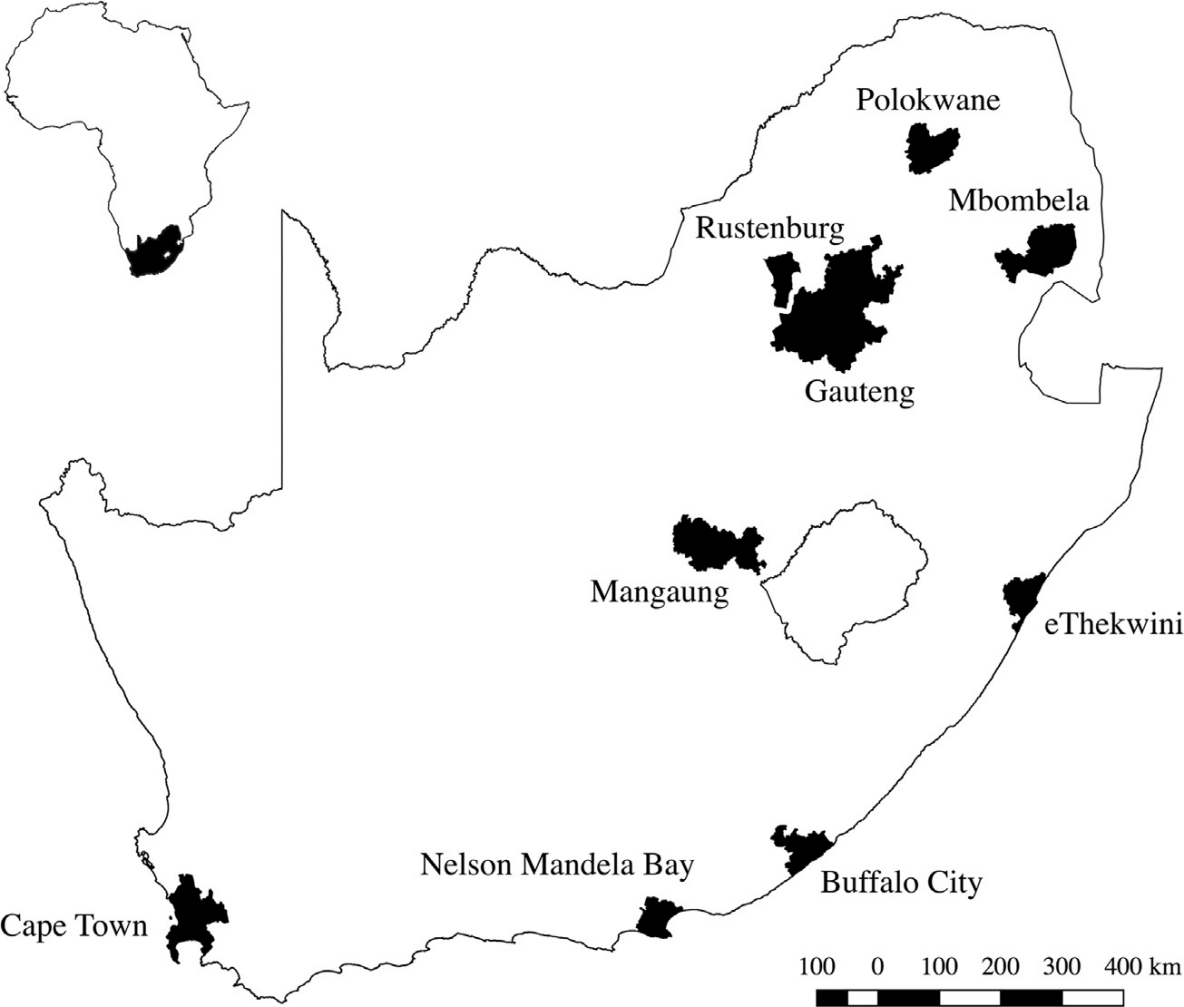
Distribution of study areas across South Africa.

The data accompanying this article include the compressed, Extensible Markup Language (XML) files of the synthetic populations for the nine areas of importance in South Africa illustrated in Fig. 1. Since there is a probabilistic component to the generation step, one hundred (100) population instances were generated for each study area. All datasets are publicly available, with the access point being *Mendeley* on https://doi.org/10.17632dh4gcm7ckb.1. The detailed XML Schema Definition (XSD) and XML Document Type Definition (DTD), which contains the declarations that describes the formal acceptable structure of the XML file, is available on http://www.matsim.org/files/dtd/. More specifically, there is one XSD definition for the household file, households_v1.0.xsd, and one DTD file for the individuals, population_v6.dtd. The files are normal XML and readable using many parsers. The choice to use the Multi-Agent Transport Simulation (MATSim) infrastructure is because the populations are frequently used for large-scale mesoscopic transport models using the agent-based MATSim [2].

## 2 Experimental design, materials and methods

### 2.1 Geographic demarcation

It is valuable to understand the spatial divisions into which South Africa was demarcated for the purpose of census enumeration [6]. The hierarchy adheres to political, administrative and statistical boundaries. At the highest, political level there is the *country*, South Africa, which is divided into nine (9) *provinces* (second political level). Then there is the *municipality*, the area of jurisdiction of the third sphere of government. At this level *municipality* refers to all three categories as defined in the Constitution, section 155.1: metropolitan (category *A*); local (category *B*); and district areas (category *C*). The *Municipal Demarcation Board* defines the boundaries of the municipalities.

In large, urban areas there is typically only category *A* metropolitan municipalities that are responsible for all municipal affairs. In smaller areas the local municipality is a defined area that is demarcated for local administrative purposes. A number of these category *B* areas make up a district municipality (category *C*), and the goal is to share resources more efficiently between the local municipalities that make up a district.

Below the municipal level there are a number of civic entities in the census hierarchy. These fall under the category *place name* and refers to easily recognizable small areas like a suburb, township or village. The first level is the *main place*, and refers to the town(ship) or city. The second level is the *subplace* that refers to the suburb or section/zone of a township. Below the subplace there are geographical working units, called *enumeration areas*, which is the lowest level of census demarcation and used for work apportioning of the fieldworkers.

Table 1 summarises the level in the demarcation hierarchy of the different study areas. Gauteng, specifically, is treated at provincial level, even though it is made up of three metropolitan municipalities (City of Tshwane, City of Johannesburg, and Ekurhuleni) and two district municipalities (Sedibeng and West Rand). The reason is because there is a lot of mobility *between* the three bordering metropolitan municipalities, making it less insightful to study these areas in isolation.

**Table 1:** The hierarchy level of the different study areas.

***Area***	***Demarcation***
Buffalo City	Metropolitan
City of Cape Town (functional)	Metropolitan
eThekwini	Metropolitan
Gauteng	Province
Mangaung	Metropolitan
Mbombela	Local municipality
Nelson Mandela Bay	Metropolitan
Polokwane	Local municipality
Rustenburg	Local municipality

Similarly, the boundaries of the City of Cape Town were extended to include the larger, functional area that incorporates the peripheral towns of Stellenbosch, Paarl, Wellington to the northeast and Malmesbury to the north.

### 2.2 Data preparation (materials)

The first phase is to prepare the raw Census data, and the following steps are required.

1. Firstly, obtain the *South African Census 2011 Community Profiles*, a SuperCROSS (proprietary) database that contains the aggregated census tables. This data is available either directly from Statistics South Africa [7], or from a public data provider like *DataFirst* [8], a research unit and data service based at the University of Cape Town, South Africa. The aggregate data accounts for the entire population and uses the *subplace* as the lowest geographic level.
  The second data set required is the 10% public use micro sample (PUMS) that is also in the public domain and is available from [9]. This data set includes the actual responses of citizens for different individual and household characteristics. To protect respondent confidentiality the data is reported at *main place* level.
2. Extract control totals from Census subplace tables for each of the nine study areas.
  a. Find the cross tabulation with *geography* (subplaces) as rows, and *income levels* from the Dwelling table as columns. There are twelve (12) income levels provided in the Census metadata (question P16_INCOME), and a 13th level denoting households who did not specify their income.
  b. Find the cross tabulation with *geography* (subplaces) as rows, and the combination of *gender* and *population group* from the Family table as columns. There are three genders specified (male, female and unspecified; question F03_SEX) but in preparing these data sets only male and female were considered. Five races (Black/African, Coloured, Indian/Asian, White and other; question P05_POP_GROUP) were considered.
  c. Merge the two tables for each study area so that rows represent the subplaces and the columns represent the three control totals: one at household and two (joint) at individual level.
3. Parse the 10% public use micro sample, filtering the individuals for each study area using the district code(s) that cover the specific study area. Since the micro sample is used as reference data to learn and estimate the socioeconomic structure, the argument for splitting the reference sample geographically is to control for structural differences between areas.

Every entry in the reference data refers to an individual. Every household has a unique household number, and each person within the household has a unique, sequential member number, where `1’ refers to the head of the household.

### 2.3 Fitting

Müller and Axhausen [3] note that the development of a synthetic population can essentially be divided into two stages. The first, *fitting*, is described in this subsection and aims to characterise the multiway distribution of all the attributes of interest by using the micro sample and marginal information available. The second stage, *generation*, is then concerned with generating a stock of individuals (linked to households) by sampling from the fitted distribution.

Table 2 lists the variables of interest that were considered in the reference data as taken from the micro sample. The interested reader is referred to Sun & Erath [11] for a critical review of different approaches to deal with the fitting problem, as it is the more complex of the two stages. In this paper Bayesian networks are employed, a promising and data-driven framework to identify causality and dependence among the set of variables.

**Table 2:** Attributes of households and individuals.

**Level**	**Variable**	**Definition (number of categories)**	**Census questionnaire reference**	**Values**
Household	Housing	Type of housing unit (6)	H01_QUARTERS	House; hostel; hotel; old age home; other; not applicable.
	Dwelling	Main dwelling type (13)	H02_DWELLINGMAIN	Formal house; traditional dwelling; apartment; cluster; townhouse; semi-detached house; formal backyard; informal backyard; informal; caravan or tent; other; unknown; not applicable.
	Rooms	Number of rooms in main dwelling (20)	H03_ROOMS	Integer values in the range [1;20].
	Tenure	The terms under which the household occupies the main dwelling (5)	H04_TENURE	Rented; owned but not yet paid off; occupied rent-free; owned and fully paid off; other.
	HhInc	The (derived) gross annual household income (in South African Rand, ZAR) (13)	P16_INCOME	0; 1–4800; 4801–9600; 9601–19,200; 19,201–38,400; 38,401–76,800; 76,801–153,600; 153,601–307,200; 307,201–614,400; 614,401–1,228,800; 1,228,801–2,457,600; 2,457,601+; Unspecified
Individual	Age	The number of completed years (birthdays celebrated) of the individual (18)	F02_AGE	0–4; 5–9; 10–14; 15–19; 20–24; 25–29; 30–34; 35–39; 40–44; 45–49; 50–54; 55–59; 60–64; 65–69; 70–74; 75–79; 80–84; 85+
	Gender	Gender of the individual (2)	F03_SEX	Male; female
	Race	Population group of the individual (5)	P05_POP_GROUP	Black/African; Coloured; Indian/Asian; White; other
	Employ	Current employment (2)	P23_EMPLOYMENTSTATUS	Yes; no
	Edu	Completed education (8)	P20_EDULEVEL	None; some primary; primary; some secondary; secondary; tertiary; other; unspecified
	Study	Current level of schooling undertaken (9)	Combination of P17_SCHOOLATTEND and P18_EDUINST	None; preschool; school; tertiary; adult education; home schooling; unknown; not applicable; unspecified

The abstract and complex relationships are extracted and presented into a simple graphical model. One advantage is that the structure of the relationships need not be defined *a priori* and imposed on the parameter estimation. Instead, the structure is learnt from the reference data, and then conditional probabilities are subsequently estimated. The implementation used in this paper closely follows that of Sun & Erath [11] and is applied independently to each of the nine study areas. A Bayesian network is estimated for each of three household types.

The firstly type is the easily identifiable, single-member households. That is, filtering the reference data on the unique household number where there is only one entry per household. Using the bnlearn library in R, the structure of the Bayesian network is learnt using a hill-climbing greedy search algorithm [5], [10]. The structure of the network is limited through what is referred to as *white-* and *blacklists*. The former are specific causal relationship links between variables that *must* exist in the network, yet the direction of the links are open for the learning algorithm to discover. In this paper there are no white-listed causal relationships used. The latter, blacklists, are specific causal relationships that are not allowed in the network. For example, there can be no causal relationship between the dwelling type and race. That is, the type of dwelling a person lives in cannot *cause* or *influence* their race. Consequently, all causal relationships that point towards the variables Age, Gender and Race, are blacklisted. This network is referred to as $g_{1}$.

The second household type considered, in terms of learning the structure of the network, are those where there is a clear household *head*, and a clear *spouse* role. Although not one of the variables of interest in the structure of the network, there is a *role* variable included in the reference data set to indicate an individual׳s relation to the household head. If, for any household, there is a *spouse* role, identifiable as the category *Husband/Wife/Partner* in the question P02_RELATION question, that household is identified as falling within this household type. The first step is to estimate a Bayesian network, denoted by $g_{21}$, which is based on the household attributes and those individual attributes of the household head and spouse. Each record in this data set represents a household of this type and one field is added, household size, n, to indicate the total number of individuals in the particular household. As for $g_{1}$, blacklisted causal relationships are again imposed. However, causal relationships between the Age, Gender and Race of the head and spouse are allowed. That is, it is argued that the Age of the household head can indeed influence the (choice of) Age of the spouse. Next a temporary data set is created that contains the rest of the household members of the households of this type. Each member makes up a record in the data set, and each record includes the personal attributes of the head, spouse, *and* the specific individual. This data set is used to train the network, denoted by $g_{22}$, with only causal relationships from the head and spouse variables to the individual׳s variables being allowed.

The third of the household types modeled are those where there is a clear household *head*, but *no spouse* role. The process is similar to the dual-role households, with the difference that the data sets only include a single head, and denote the two networks are denoted as $g_{31}$ for the head and $g_{32}$ for the rest of the household members, respectively.

Although the structure of the different Bayesian networks estimated can yield interesting insight into the population, their interpretation falls outside the scope of this paper. The interested reader is referred to Sun & Erath [11] for illustrative examples.

### 2.4 Generation

With the structure of the Bayesian networks for the three household types known, the next step is to generate a pool of households for each of the study areas. The distributions of the three household types differ for the different areas, and are shown in Table 3 along with the size of the household pool that was simulated. The number to simulate for each area is approximately 20% of the estimated population size (in 2011, the reference date for the Census) and is based on the suggestion by Sun & Erath [11] and their population for Singapore.

**Table 3:** Distribution of the three household types.

**Study area**	**Household type**			**Individual observations in reference data**	**Pool size simulated**
	**Single member**	**Dual role**	**Single role**		
Buffalo City	7.0%	46.9%	46.1%	62,663	120,000
City of Cape Town	5.1%	63.0%	31.9%	380,739	750,000
eThekwini	6.0%	48.6%	45.4%	268,374	500,000
Gauteng	8.4%	55.8%	35.8%	977,475	2,000,000
Mangaung	6.3%	52.1%	41.6%	64,351	150,000
Mbombela	5.4%	42.8%	51.8%	140,696	300,000
Nelson Mandela Bay	4.8%	54.4%	40.8%	97,634	200,000
Polokwane	6.1%	38.1%	55.8%	108,486	250,000
Rustenburg	9.1%	49.2%	41.7%	121,104	250,000

Sampling from the individual member household network, $g_{1}$, is straightforward, and the bnlearn library in R is used [10]. The number of individuals to sample is based on the study area׳s fraction for single-member households. For example, in Buffalo City $s_{1}=0.07\left(120000\right)=8400$ individuals are sampled. For the dual role households, the process is different. The total number of *individuals* for this household type should be, using Buffalo City as an example again, at least $s_{2}\geq 0.469\left(120000\right)=56280$. First the head and spouse are sampled from $g_{21}$, which also gives the household size, n. Then the household is completed by sampling n−2 individuals from $g_{22}$, conditional to the attributes of the household head and spouse sampled. Sampling of complete households is repeated in this way until the total number of individuals is greater than or equal to $s_{2}$.

A similar procedure is used for households with a single head role. The household head is sampled from $g_{31}$, along with the household size n. The rest of the n−1 household members are then sampled from $g_{31}$. This is repeated until the overall population pool size is reached.

One of the benefits of using Bayesian networks to generate a large pool is that one is able to synthesize households and household structures that were not necessarily observed in the 10% PUMS. With the pool of households and individuals generated uniquely for each study area, the households for the different sub places are sampled. This is done through the generalized raking method of survey sampling to first get the probability (or weight) of each household in the pool appearing in the particular sub place [1]. The weights are generated for each sub place using the control totals of both the households and the individuals. Since the sampling is random, 100 different synthetic populations can be generated – using the same probability set for each sub place – by simply setting the random seed. For the generalized raking procedure the implementation of Mueller [4] is used.

## 3 Population container

The output of this procedure is a complete stock of individuals that are arranged in their households. These are then parsed using the MATSim infrastructure that includes linked containers for households and individuals.

**Fig. 2:**
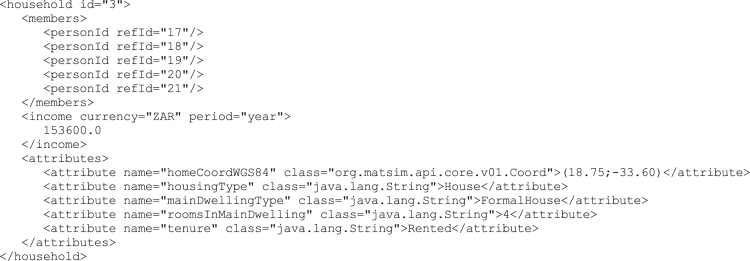
Example excerpt from the final household XML file.

The households are numbered consecutively, starting from zero, with no particular order. An example of a five-member household in the XML format is shown in Fig. 2. The example is taken from population 10 of the City of Cape Town scenario. Each household has a unique identifying number (id).

All the household members are listed by their own unique identifying number (personId). The household׳s income is then expressed by the upper value of the income band as given in the Census categories. It is worth noting that the currency (South African Rand, ZAR) is expressed in this version of the data (version 1) in 2011 value. For each household the main attributes, as taken from the variables in Table 2, are listed. One additional attribute is added, namely the household׳s home coordinate, expressed in decimal degrees using the latest version (1984) of the World Geodesic System (WGS84). The home coordinate is a randomly sampled point inside the subplace from which the household originates. The subplace shapefiles are distributed with the Census data.

Each attribute is listed by it׳s name, and then by its specific Java class type. For example, the housingType is a standard Java string, while the homeCoordWGS is a specific coordinate class in the MATSim open source project [2].

**Fig. 3:**
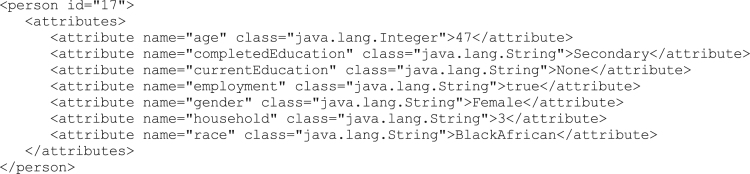
Example excerpt from the final population XML file.

Each household member listed in the household refers to a unique individual in the population.xml.gz file. If the household shown in Fig. 2 is used as an example, looking at the first member with personId 17, the individual is shown in Fig. 3.

In this example it is a 47-year old, black African female who completed secondary education (high school in South Africa); is currently employed; not participating in any educational activity at this point; and there is a back-pointer to her household identifier, 3.

The same household (population 10 from City of Cape Town) has four other members, and what follows is an interpretation of their attributes. The single mother-led household includes a younger black African couple made up of an unemployed, 27-year old male (person 18) with only a partial secondary education, and an unemployed, 27-year old female (person 19) with completed secondary education. The young couple has two children – both boys – one an infant (person 20) and the other a 7-years old who is currently in primary school (person 21).

## References

[1] Deville J.-C., Särndal C.-E., Sautory O. (1993). Generalized raking procedures in survey sampling. J. Am. Stat. Assoc..

[2] Horni A., Nagel K., Axhausen K.W. (2016). The Multi-Agent Transport Simulation MATSim.

[3] K. Mueller, K.W. Axhausen, Population synthesis for microsimulation: state of the art, in: Transportation Research Board 90th Annual Meeting. Washington, D.C., 2011.

[4] K. Mueller, MultiLevelIPF: Implementation of algorithms that extend IPF to nested structures. Available online from 〈https://github.com/krlmlr/MultiLevelIPF〉, 2018.

[5] R Core Team (2017). R: A Language and Environment for Statistical Computing. https://www.R-project.org/.

[6] Statistics South Africa, Census 2011 Metadata. Report No 03-01-46. Pretoria, South Africa, 2012.

[7] Statistics South Africa, South African Census Community Profiles 2011. Available online from 〈http://www.statssa.gov.za/?Page_id=3955〉, 2015.

[8] Statistics South Africa (2015). South African Census Community Profiles 2011 [data set]. Version 1. Pretoria: Statistics South Africa [producer], 2014.

[9] Statistics South Africa (2015). South African Census 2011 10% Sample [data set]. Version 2. Pretoria: Statistics South Africa [producer], 2015.

[10] Scutari M. (2010). Learning Bayesian Networks with the bnlearn R package. J. Stat. Softw..

[11] Sun L., Erath A. (2015). A Bayesian network approach for population synthesis. Transp. Res. Part C.

